# Hunting out the repeat expansion in Huntington’s pigs

**DOI:** 10.1093/procel/pwad014

**Published:** 2023-03-28

**Authors:** Guang Yang, Boxun Lu

**Affiliations:** Neurology Department at Huashan Hospital, State Key Laboratory of Medical Neurobiology and MOE Frontiers Center for Brain Science, School of Life Sciences, Fudan University, Shanghai 200438, China; Neurology Department at Huashan Hospital, State Key Laboratory of Medical Neurobiology and MOE Frontiers Center for Brain Science, School of Life Sciences, Fudan University, Shanghai 200438, China

A recent study from Yan et al. (2023) provides the milestone evidence in the large animal model demonstrating genome editing as a potential therapeutic strategy for Huntington’s disease treatment.

Huntington’s disease (HD) is a devastating monogenetic neurodegenerative disorder that is caused by an expanded CAG repeat in the exon 1 of the *HTT* gene located in the 4th chromosome of the human genome (Macdonald, 1993). The prevalence of HD is ~1 out of 10,000 and carrier of the pathogenic mutant gene is many times more than this (Gardiner et al., 2019). Thus, HD may potentially influence more than several millions of people. The average onset age of HD is typically 40–50 years old, and premature death often occurs 10–15 years later. HD is characterized by progressive neurodegeneration mainly in the striatum, leading to severe clinical symptoms including chorea, which is a disorder exhibiting involuntary, irregular, unpredictable movements. Currently there are only symptom relief medications but no disease progression-modifying drugs, leading to a tremendous unmet medical need for a fundamental treatment approach of HD. In a recent paper published in *Nature Biomedical Engineering* by Yan et al., scientists made an extraordinary advancement towards the ultimate cure of HD.

Given its monogenetic nature, gene therapy that targets the mutant gene (*HTT*) or its transcript has been considered as promising strategies for HD treatment. For example, anti-sense oligonucleotides (ASOs) that suppress the *HTT* mRNA have been investigated in the HD mouse models and then entered the clinical trials (Tabrizi et al., 2019). The clinical efforts have been unsuccessful though, due to many possible reasons. One thing to note is that the preclinical studies of ASOs were mainly carried out using the HD mouse models, which lacked the typical degeneration of medium spiny neurons in the striatum seen in the HD patients and exhibited relatively mild motor functional deficits. Thus, HD animal models that exhibit selective neurodegeneration and more patient-like behavioral phenotypes are likely critical for preclinical assessment of HD treatment strategies. A previous study from the same group established an HD knock-in pig model that mimicked both the HD genetic mutation and phenotypes faithfully (Yan et al., 2018). The model expresses an endogenous mutant human *HTT* gene harboring 150×CAG repeats, and displays striking and selective degeneration of striatal medium spiny neurons, providing a great tool for preclinical HD studies of gene therapy approaches beyond rodent models and enabling the current study from this group.

**Figure 1.**
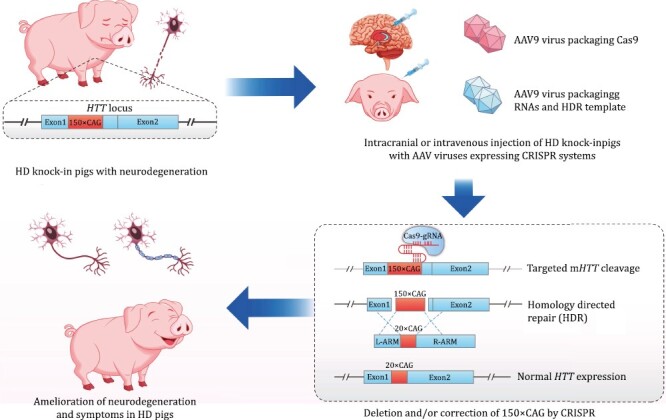
Scheme of correction and ameliorated symptoms of Hungtington’s disease in pig model with CRISPR system-mediated gene therapy.

In their most recent study published in *Nature Biomedical Engineering* (Yan et al., 2023), Yan et al. used the CRISPR-mediated gene knock-in approach to correct the pathogenic 150×CAG repeat expansion to the normal 20×CAG repeats in the mutant *HTT* DNA to tackle the disease (Fig. 1). Different from the ASOs that target the *HTT* RNA and reduce its expression, CRISPR is a genome editing approach targets the DNA directly to create permanent modifications. It utilizes a guided RNA (gRNA) to navigate a nuclease (Cas9) to the target genome site and cleave it, triggering genome editing by generating double-stranded breaks (DSBs) and the subsequent DNA repair. CRISPR has been applied in both plants and animals, including rodents and non-human primates (NHP) (Hsu et al., 2014). Importantly, correction or deletion of pathogenic mutations by CRISPR showed great promises in curing several genetic diseases such as blindness and sickle cell disease (SCD) based preclinical studies and entered clinical trials (Ledford, 2020; Zeng et al., 2020). Recently, CRISPR-based new technologies further increased the targeting scope and could treat more specific genetic diseases. For example, base editing (BE) can change a single base pair to correct a point mutation (C·G→T·A and A·T→G·C substitutions) precisely, and prime editing (PE) can insert, delete, or mutate all point mutations (C·G→T·A, A·T→G·C, C·G→A·T, C·G→G·C, A·T→T·A, A·T→C·G). These emerging technologies provide powerful new tools to cure genetic diseases caused by different categories of genetic mutations (Rees and Liu, 2018; Anzalone et al., 2019).

In the Yan et al. study, the authors used their previous established HD KI pig model harboring 150×CAG repeats to test the potential therapeutic effects of genome editing by the CRISPR-mediated knock-in approach. They used the adeno-associated virus (AAV) to package the CRISPR elements, including Cas9, gRNAs, and the homologous recombination template for m*HTT* correction. They applied the AAVs to the striatum of HD KI pigs by stereotaxic injection, and the injected AAVs successfully “hunt out” the CAG repeat expansion with remarkably high efficiency and corrected it with a normal 20×CAG by homology directed repair (HDR) following CRISPR-mediated DNA cleavage. This genetic correction led to unprecedented phenotypic rescue that has never been observed in HD animal models previously. At the molecular level, the authors detected a correction of the transcriptome towards the wild-type (WT) after AAV injection, and the expression of mutant HTT protein (mHTT) was also drastically decreased. At the cellular level, the *in vivo* neurodegeneration and glia activation were significantly ameliorated. At the whole animal level, the injected pigs exhibited alleviated HD-relevant symptoms such as gait abnormalities and shortened lifespan. Overall, the study demonstrates that CRIPSR-mediated gene correction is an extremely promising new approach to fundamentally treat HD.

More specifically, there are several highlights of this study: (i) The gene therapy of HD in large animal models. Almost all the HD gene therapy studies were performed in mouse model, which typically lacked obvious neurodegeneration to mimic human HD. The pig models exhibit HD-like phenotypes with higher fidelity and are much closer to the patient’s symptoms, providing an ideal tool for HD therapeutic studies; (ii) Remarkable therapeutic effects after the gene editing by a single intracranial or intravenous injection of AAVs. Unlike the ASO-based gene therapy approaches that require multiple intrathecal injections per year, this study showed that a single peripheral injection could potentially treat HD, providing a much more desirable scenario for potential clinical application; (iii) Potential safety issues were systematically evaluated, including deep-sequencing and whole-genome sequencing that reveal minimal off-target effects and strong animal safety data that encourages further clinical trials.

Scientists have been seeking for a fundamental HD therapy for decades without a final success. The Yan et al. study provides a great new hope for the HD patients by leading a new orientation of HD treatment approach. This promising study also inspires many future studies. The CAG repeat replacement is very promising but its efficiency remains to be improved. Besides potential clinical trials utilizing the reported approach, other genome-editing technologies could be considered to tackle HD or further improve the current approach. In light of the recent study showing that CRISPR may lead to endogenous DNA damage response arising from aberrant *p53* activation (Enache et al., 2020), inactivated Cas9-based CRISPR methods such as dCas9, base editing, or prime editing, could be considered to further improve safety. Ideally, they should be tested in the pig models as well. Finally, the Yan et al. study also provides the proof-of-concept evidence that justifies mHTT-lowering as a potential HD therapeutic approach. Thus, small molecule compounds that may lower mHTT levels (Li et al., 2019) are also worth further optimization and investigation, ideally in the pig models.
